# Supplementary material for: Profiling of Breast Cancer Stem Cell Types/States Shows the Role of CD44hi/CD24lo-ALDH1hi as an Independent Prognostic Factor After Neoadjuvant Chemotherapy
Source: Int J Mol Sci. 2025 Aug 24;26(17):8219. doi: 10.3390/ijms26178219 (PMC12428598; doi:10.3390/ijms26178219)
Supplement: Supplementary file 1 [file ijms-26-08219-s001.zip › Supplementary Table S1.pdf]

Supplementary Table S1. Patient Characteristics.

Features	Categories	# Patients
**Age**	< 40 years<br>≥ 40 years	32 *(39)<br>51 (61)
**Tumor Size**	< 4 cm<br>≥ 4 cm	39 (53)<br>34 (47)
**Histological Grade**	Grade 1<br>Grade 2<br>Grade 3	2 (3)<br>35 (48)<br>36 (49)
**Lymph node involvement**	Absent<br>Present	26 (36)#<br>46 (64)
**Estrogen Receptor (ER)**	Negative<br>Positive	27 (37)<br>46 (63)
**Progesterone Receptor (PR)**	Negative<br>Positive	40 (55)<br>33 (45)
**HER2/neu**	Negative<br>Positive	49 (67)<br>24 (33)
**Subtype**	Luminal A<br>Luminal B<br>Her2<br>TNBC	25 (34)<br>21 (29)<br>12 (16)<br>15 (21)
**Neoadjuvant Chemotherapy**	None<br>♠AC only<br>FAC regimen<br>AC + Docetaxel<br>Other regimens	30 (41)<br>7 (10)<br>21 (29)<br>9 (12)<br>6 (8)

* Percentage of cases
# 1 case unknown status
♠ AC= Adriamycin (doxorubicin) + Cyclophosphamide
FAC= Fluorouracil + Adriamycin + Cyclophosphamide
