# Supplementary material for: Profiling of Breast Cancer Stem Cell Types/States Shows the Role of CD44hi/CD24lo-ALDH1hi as an Independent Prognostic Factor After Neoadjuvant Chemotherapy
Source: Int J Mol Sci. 2025 Aug 24;26(17):8219. doi: 10.3390/ijms26178219 (PMC12428598; doi:10.3390/ijms26178219)
Supplement: Supplementary file 1 [file ijms-26-08219-s001.zip › Supplementary Table S2.pdf]

Supplementary Table S2. Correlation between different CSCs states/types and immune/ proliferation markers (**A**) or clinicopathological parameters (**B**) in 73 breast cancer patients.

A

		CD24^{hi}			§CD10^{hi}			BMI1^{hi}		
		H-Score <140	H-Score ≥140	♣*p*	< 5%	≥ 5%	*p*	Score 0/1	Score 2/3	*p*
Immune Markers	**PD-L1**									
	< 5%	**43 (73)***	**16 (27)**	**0.013**	49 (86)	8 (14)	0.444	41 (72)	16 (28)	0.745
	≥ 5%	**5 (36)**	**9 (64)**		11 (79)	3 (21)		11 (79)	3 (21)	
	**TIL**									
	Low (Score 1 & 2)	41 (72)	16 (28)	0.071	48 (87)	7 (13)	0.253	38 (69)	17 (31)	0.205
	High (Score 3)	7 (44)	9 (56)		12 (75)	4 (25)		14 (88)	2 (12)	
	**FOXP3+TIL**									
	< 10%	**31 (84)**	**6 (16)**	**0.001**	33 (89)	4 (11)	0.306	**23 (62)**	**14 (38)**	**0.008**
	≥ 10%	**12 (43)**	**16 (57)**		22 (79)	6 (21)		**26 (93)**	**2 (7)**	
	§ **PD-1+TIL**									
	< 10%	**26 (81)**	**6 (19)**	**0.012**	48 (87)	4 (13)	0.533	23 (72)	9 (28)	0.786
	≥ 10%	**19 (50)**	**19 (50)**		31 (82)	7 (18)		29 (76)	9 (24)	
Proliferation Markers	**Ki-67**									
	≤ 20%	**28 (78)**	**8 (22)**	**0.048**	33 (92)	3 (8)	0.111	23 (64)	13 (36)	0.107
	> 20%	**20 (54)**	**17 (46)**		27 (77)	8 (23)		29 (83)	6 (17)	
	**SKP2**									
	< 10%	**36 (78)**	**10 (22)**	**0.002**	40 (89)	5 (11)	0.173	29 (64)	16 (36)	0.051
	≥ 10%	**10 (40)**	**15 (60)**		18 (75)	6 (25)		21 (88)	3 (12)	
	**p21**									
	< 10%	24 (56)	19 (44)	0.075	36 (86)	6 (14)	0.740	34 (81)	8 (19)	0.059
	≥ 10%	22 (79)	6 (21)		22 (81)	5 (19)		16 (59)	11 (41)	
	**p27**									
	< 50 %	30 (59)	21 (41)	0.107	40 (80)	10 (20)	0.270	**42 (84)**	**8 (16)**	**0.002**
	≥ 50 %	16 (80)	4 (20)		18 (95)	1 (5)		**8 (42)**	**11 (58)**	
	**Loss of both p21/p27**									
	NO	**15 (48)**	**16 (52)**	**0.014**	24 (80)	6 (20)	0.514	**27 (90)**	**3 (10)**	**0.006**
	YES	**31 (77)**	**9 (23)**		34 (87)	5 (13)		**23 (59)**	**16 (41)**	

B

		CD24^{hi}			CD10^{hi}			BMI1^{hi}		
		H-Score <140	H-Score ≥140	*p*	< 5%	≥ 5%	*p*	Score 0/1	Score 2/3	*p*
Clinicopathological Markers	**Age**									
	< 40 years	16 (67)	8 (33)	1.000	19 (86)	3 (14)	1.000	15 (68)	7 (32)	0.569
	≥ 40 years	32 (65)	17 (35)		41 (84)	8 (16)		37 (76)	12 (24)	
	**Tumor Size**									
	< 4 cm	26 (67)	13 (33)	1.000	34 (87)	5 (13)	0.527	**23 (59)**	**16 (41)**	**0.003**
	≥ 4 cm	22 (65)	12 (65)		26 (81)	6 (19)		**29 (91)**	**3 (9)**	
	**Invasion**									
	Absent	17 (57)	13 (43)	0.214	24 (83)	5 (17)	0.750	22 (76)	7 (24)	0.788
	Present	31 (72)	12 (28)		36 (86)	6 (14)		30 (71)	12 (29)	
	**Histological Grade**									
	1 & 2	**30 (81)**	**7 (19)**	**0.007**	31 (89)	4 (11)	0.514	22 (63)	13 (37)	0.064

	3	**18 (50)**	**18 (50)**		29 (81)	7 (19)		30 (83)	6 (17)	
	**§Lymph Node Metastasis**									
	Absent	17 (65)	9 (35)	1.000	22 (88)	3 (12)	0.735	20 (80)	5 (20)	0.405
	Present	31 (67)	15 (33)		37 (82)	8 (18)		31 (69)	14 (31)	
	**Neoadjuvant Chemotherapy**									
	Absent	22 (73)	8 (27)	0.449	25 (83)	5 (17)	1.000	22 (73)	8 (27)	1.000
	Present	27 (63)	16 (37)		35 (85)	6 (15)		30 (73)	11 (27)	
BC Subtype Markers	**ER Status**									
	Negative	**12 (44)**	**15 (56)**	**0.005**	19 (73)	7 (27)	0.085	**23 (88)**	**3 (12)**	**0.049**
	Positive	**36 (78)**	**10 (22)**		41 (91)	4 (9)		**29 (64)**	**16 (36)**	
	**PR Status**									
	Negative	23 (58)	17 (42)	0.138	30 (77)	9 (23)	0.096	30 (77)	9 (23)	0.591
	Positive	25 (76)	8 (24)		30 (94)	2 (6)		22 (69)	10 (31)	
	**Her2/neu Status**									
	Negative	**37 (76)**	**12 (25)**	**0.018**	42 (89)	5 (11)	0.165	32 (68)	15 (32)	0.258
	Positive	**11 (46)**	**13 (54)**		18 (75)	6 (25)		20 (83)	4 (17)	
	**TNBC Status**									
	Negative	41 (71)	17 (29)	0.125	48 (84)	9 (16)	1.000	40 (70)	17 (30)	0.324
	Positive	7 (47)	8 (53)		12 (86)	2 (14)		12 (86)	2 (14)	
EMT Markers	**Vimentin**									
	Negative	**38 (75)**	**13 (25)**	**0.030**	43 (86)	7 (14)	0.721	35 (70)	15 (30)	0.395
	Positive	**10 (45)**	**12 (55)**		17 (81)	4 (19)		17 (81)	4 (19)	
	**Loss of E-Cadherin**									
	Negative	33 (65)	18 (35)	1.000	43 (86)	7 (14)	0.721	36 (72)	14 (28)	0.778
	Positive	15 (68)	7 (32)		17 (81)	4 (19)		16 (76)	5 (24)	
	**Vimentin/Loss of E-Cadherin**									
	Negative	42 (68)	20 (32)	0.494	53 (87)	8 (13)	0.180	44 (72)	17 (28)	0.719
	Positive	6 (55)	5 (45)		7 (70)	3 (30)		8 (80)	2 (20)	

Abbreviations: *(+ and -) are the number of positive and negative patients. The numbers in brackets are the percentages of patients, and ♣*p* values in bold and shaded represent significant data. Light shading represents borderline significance. **A)** § CD10 and BMI1 is unknown for two samples and PD-1 is unknown for 3 samples. **B)** BMI1 and CD10 is unknown for 2 cases.
