# Supplementary figures and images for: Profiling of Breast Cancer Stem Cell Types/States Shows the Role of CD44hi/CD24lo-ALDH1hi as an Independent Prognostic Factor After Neoadjuvant Chemotherapy
Source: Int J Mol Sci. 2025 Aug 24;26(17):8219. doi: 10.3390/ijms26178219 (PMC12428598; doi:10.3390/ijms26178219)

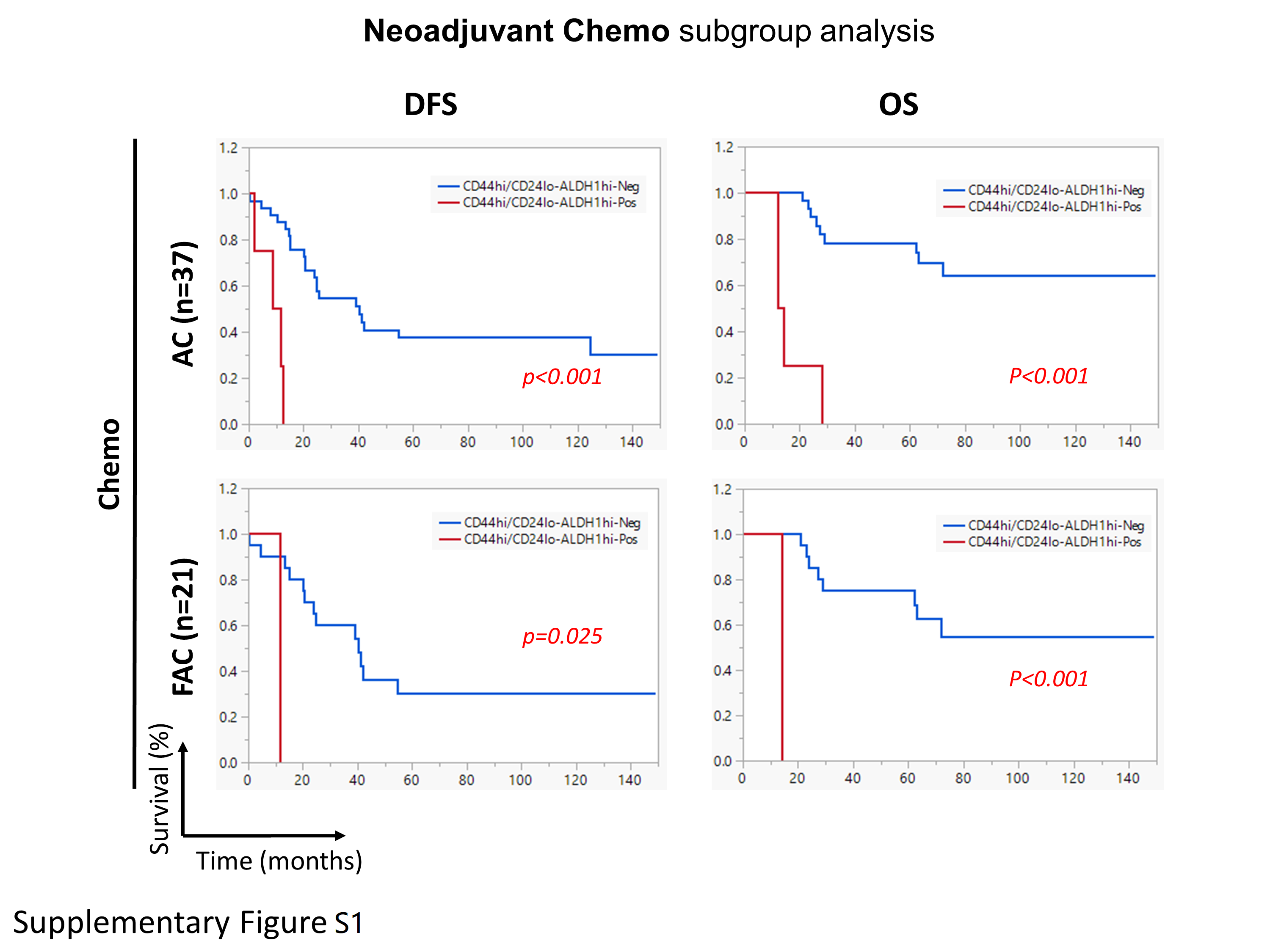

Supplement: Supplementary file 1 [file ijms-26-08219-s001.zip › Supplementary Figure S1.tif]

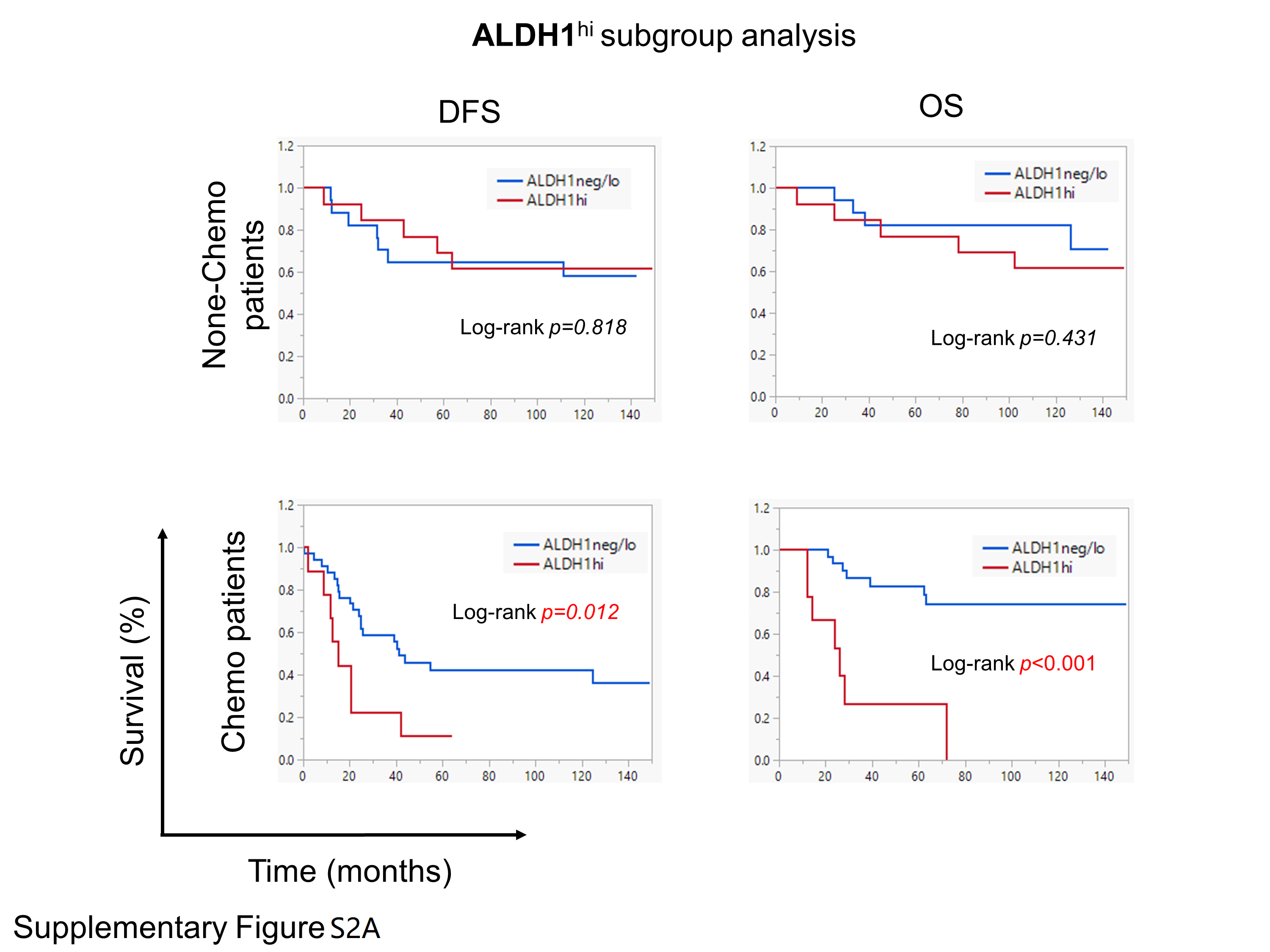

Supplement: Supplementary file 1 [file ijms-26-08219-s001.zip › Supplementary Figure S2A.TIF]

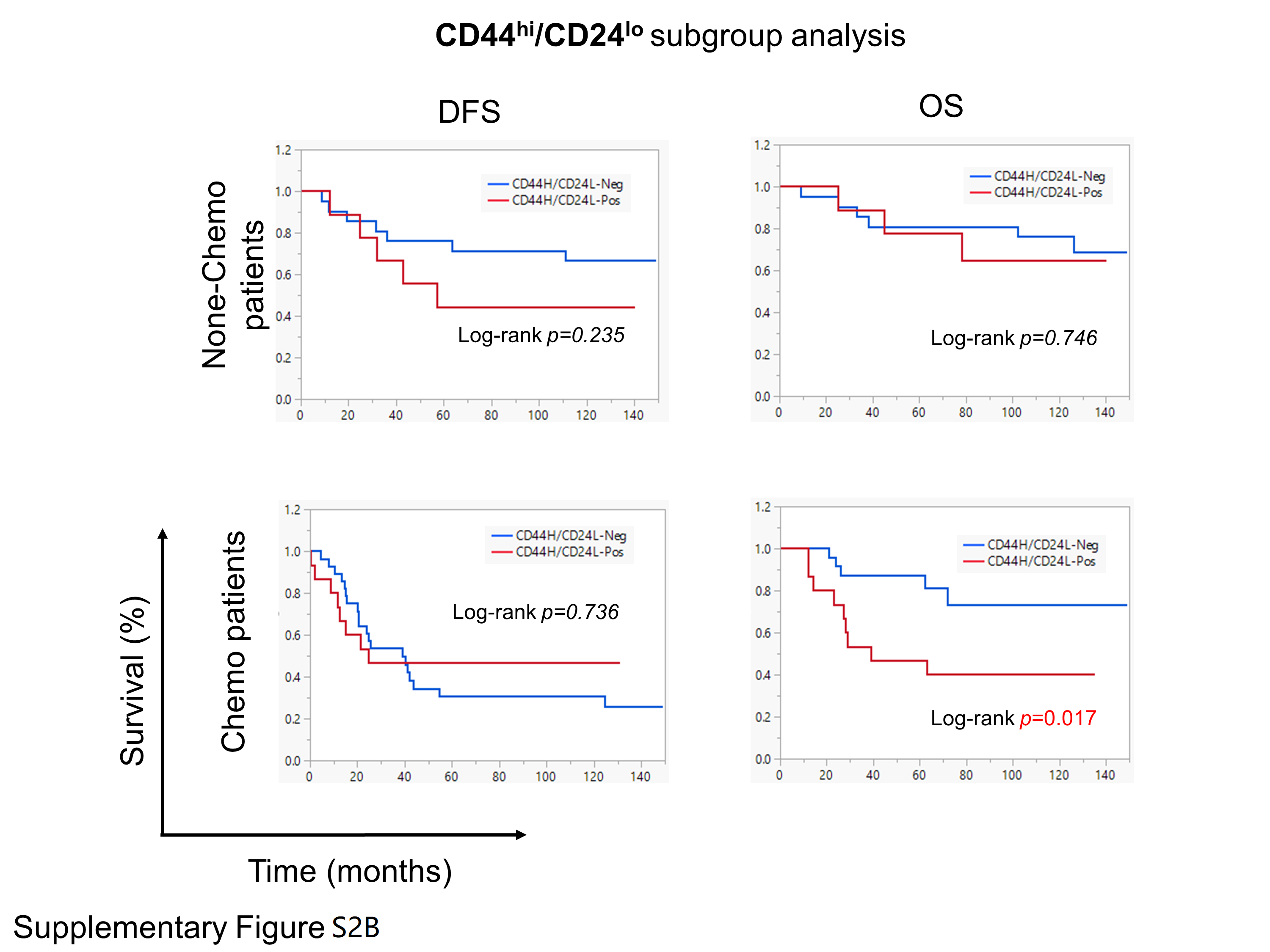

Supplement: Supplementary file 1 [file ijms-26-08219-s001.zip › Supplementary Figure S2B.TIF]
